# Spheroidal Model of SKBR3 and U87MG Cancer Cells for Live Imaging of Caspase-3 during Apoptosis Induced by Singlet Oxygen in Photodynamic Therapy

**DOI:** 10.3390/biomedicines10092141

**Published:** 2022-08-31

**Authors:** Viktória Pevná, Mariana Máčajová, Andrej Hovan, Gregor Bánó, Majlinda Meta, Boris Bilčík, Júlia Palková, Veronika Huntošová

**Affiliations:** 1 Department of Biophysics, Institute of Physics, Faculty of Science, P.J. Safarik University in Kosice, Jesenna 5, 041 54 Kosice, Slovakia; 2 Institute of Animal Biochemistry and Genetics, Centre of Biosciences, Slovak Academy of Sciences, Dubravska cesta 9, 840 05 Bratislava, Slovakia; 3 Center for Interdisciplinary Biosciences, Technology and Innovation Park, P.J. Safarik University in Kosice, Jesenna 5, 041 54 Kosice, Slovakia

**Keywords:** hypericin, spheroid, singlet oxygen, photodynamic therapy, caspase-3, apoptosis, cancer, imaging

## Abstract

Aspects related to the response of cells to photodynamic therapy (PDT) have been well studied in cell cultures, which often grow in monolayers. In this work, we propose a spheroidal model of U87MG and SKBR3 cells designed to mimic superficial tumor tissue, small spheroids (<500 µm) suitable for confocal fluorescence microscopy, and larger spheroids (>500 µm) that can be xenografted onto quail chorioallantoic membrane (CAM) to study the effects of PDT in real time. Hypericin was used as a model molecule for a hydrophobic photosensitizer that can produce singlet oxygen (^1^O_2_). ^1^O_2_ production by hypericin was detected in SKBR3 and U87MG spheroid models using a label-free technique. Vital fluorescence microscopy and flow cytometry revealed the heterogeneity of caspase-3 distribution in the cells of the spheroids. The levels of caspase-3 and apoptosis increased in the cells of spheroids 24 h after PDT. Lactate dehydrogenase activity was evaluated in the spheroids as the most reliable assay to detect differences in phototoxicity. Finally, we demonstrated the applicability of U87MG spheroids on CAM in photodiagnostics. Overall, the variability and applicability of the prepared spheroid models were demonstrated in the PDT study.

## 1. Introduction

Photodynamic therapy (PDT) is a treatment method that requires minimal injury to cause cancer cell death. It consists of three main components: photosensitizer, oxygen, and light of an appropriate wavelength corresponding to the absorption parameters of the photosensitizer. The processes induced by PDT in cells often lead to apoptosis or another type of cell death, depending on the photosensitizer used [1].

The generation of reactive oxygen species during PDT is well known. One of the most demanding of them is singlet oxygen. This highly reactive form has a short lifetime and diffusion radius due to its rapid deactivation and reactivity with biological material [2]. Singlet oxygen can activate caspase-3, protein phosphatases, p38 mitogen activation of protein kinases, and release of cytochrome c from mitochondria [3,4].

In the present study, hypericin was used as a model molecule for a hydrophobic photosensitizer. It has very interesting photodynamic and photodiagnostic properties [5]. Moreover, it has a dual character. Its monomer emits a bright fluorescence with a maximum at 600 nm, which can be easily detected in photodiagnostics [6]. In contrast, its aggregated form, which occurs in aqueous solutions, is non-fluorescent and ineffective in PDT [7,8]. Hypericin, a natural compound, has been presented as a very potent photosensitizer that induces PDT via singlet oxygen generation [9]. It has been shown to induce apoptosis in several cell lines, with p38, Jun N terminal kinase, caspase-9, and caspase-3 playing important roles [10,11,12,13].

The characterization of cell death depends heavily on cell physiology, and most methods to quantify and identify the nature of cell death have been developed in vitro and mainly in monolayers of cell cultures [14]. In recent years, great efforts have been made to model tumors. For this reason, the development of organoids and spheroids has been given high priority [15,16]. Multicellular 3D cultures can be prepared by various techniques, such as the hanging drop method, scaffold-based and scaffold-free techniques, which require Matrigel and Methocel additives for further growth and development [17,18].

It has been reported that the permeation of hypericin in spheroids correlates with E-cadherin expression. The estimation of parameters determining the effect of PDT strongly depends on the morphology of spheroids, and differs from those obtained in monolayers of cells [19,20,21,22,23].

In the present work, scaffold-free and additive-free models of cancer cells are presented in three types of spheroids (superficial, small (<500 µm) and large (>500 µm) spheroids) suitable for label-free ^1^O_2_ detection and real-time monitoring of caspase-3 after hypericin-induced PDT. Spheroids represent structures in which metabolic activity, oxygen and nutrient supply are heterogeneously distributed. For this reason, it can be assumed that ^1^O_2_ production and cell response to PDT are also heterogeneous. The main goal of this study was to highlight the experimental limitations arising from the 3D structure of the spheroid models. Confocal fluorescence microscopy and flow cytometry were used here to assess the heterogeneity of apoptosis and caspase-3 in human glioblastoma cells U87MG, grown in spheroids and in human ductal carcinoma SKBR3 prepared as a compact aggregated (superficial) 3D model. Time-resolved phosphorescence measurements were used to determine the availability of ^1^O_2_ production in spheroids. The applicability of U87MG spheroids in the hanging drop model for in vivo photodiagnostic applications was demonstrated using a model of quail embryonic chorioallantoic membrane (CAM).

## 2. Materials and Methods

### 2.1. Cell Cultures and Spheroids Preparation

The human breast cancer cell line SKBR 3 (a gift from the laboratory of Prof. Pluckthun, University of Zurich, Switzerland) was grown in RPMI 1640 (LM-R1638/500, Biosera, Nuaille, France). The human glioma cells U87MG (Cells Lines Services, Eppelheim, Germany) were grown in DMEM (Dulbecco’s modified Eagle medium, high glucose, GlutaMAXTM, with pyruvate, Gibco-Invitrogen, Life Technologies Ltd., Paisley, UK). Cell culture media were supplemented with 10% FBS (fetal bovine serum, Gibco-Invitrogen, Life Technologies Ltd., Paisley, UK) and 1% (*w*/*w*) penicillin/streptomycin (Gibco-Invitrogen, Life Technologies Ltd., Paisley, UK). Cells were grown in the dark under a humidified atmosphere, 5% CO_2_, and 37 °C. Two types of protocols were used in the experiment: the hanging drop method and spontaneously growing spheroids.

Hanging drop method: after reaching 80% confluence, U87MG or SKBR3 cells were centrifuged and seeded (5 × 10^4^ cells per droplet) upside down in 50 µL droplets on Petri dishes caps to form spheroids for 4 days. Spheroids were then transferred to a fresh complete (containing 10% FBS) cell culture medium where they were allowed to grow further. Upon reaching a diameter of 1 mm, the spheroids were then used in experiments. While U87MG cells formed nice uniform spheroids, SKBR3 cells formed compact 3D aggregates.

Spontaneously growing spheroids: U87MG cells were seeded at a density of 10^6^ cells per flask and allowed to grow for two weeks. The cell culture medium was changed every 4 days. On the day of the experiment, the spheroids were removed and seeded in a fresh complete cell culture medium.

### 2.2. Photodynamic Treatment of the Spheroids

The cells were treated with 500 nM hypericin dissolved in 100% dimethyl sulfoxide (DMSO, Sigma-Aldrich, St. Louis, MO, USA) for 3 h. The final amount of DMSO added to the cell culture medium was less than 0.1%. A control with vehicle (DMSO) was performed, but it did not affect the results and gave the same results as the control without DMSO. The medium containing hypericin was removed and replaced with a complete cell culture medium. Subsequently, these cells were irradiated with 590 nm light-emitting diodes (home-made system) for 2, 4, 6, and 10 min, corresponding to light doses of 2, 4, 6, and 10 J/cm^2^. The response of the cells to PDT was determined 24 h after PDT. The optimization of the therapeutic protocol was based on our previous publication, in which the control measurements were performed without hypericin and light [12,24]. No significant effects of light application were observed in the studied cells.

### 2.3. Imaging of Spheroids

RGB color images of spheroids were captured with the color camera of the Huawei P30 Pro Android smartphone (Huawei Technologies Co., Ltd., Shenzhen, China). Microscopic images of spheroids were acquired using a confocal fluorescence microscope system (LSM 700, Zeiss, Oberkochen, Germany), FLUAR 20×/0.75 and Plan-NEOFLUAR 5×/0.15 objectives (Zeiss), and a CCD camera (AxioCam HRm, Zeiss). The fluorescence images were analyzed using Zen 2011 software (Zeiss). Fluorescence was excited either with solid-state lasers at 405 nm, 488 nm, and 555 nm or with light from the mercury-tungsten lamp filtered with the filter cube FS10 (ZEISS, excitation BP 450–490, emission BP 515–565) for the NucView^®^488 caspase-3 substrate (biotium, Fremont, CA, USA) and FS15 (ZEISS, excitation BP 546/12, beam splitter FT 580, emission LP 590) for hypericin in SKBR3 spheroids.

Nuclei were labeled with 10 µg/mL Hoechst 33258 (ThermoFisher Scientific, Waltham, MA, USA) for 30 min (excitation at 405 nm and emission in the 450 ± 40 nm spectral range). NucView^®^488 caspase-3 substrate was used to monitor caspase-3 level in cells (excitation at 488 nm and emission in the spectral range of 490–560 nm). Hypericin was detected in the spectral range >590 nm after excitation at 555 nm.

### 2.4. Assessment of Phototoxicity in 3D Spheroids

Detection of phototoxicity associated with changes in cellular/mitochondrial metabolism was performed according to the supplier’s protocol and 24 h after treatment. In this assay, 3-(4,5-dimethylthiazol-2-yl)-2,5-diphenyltetrazolium bromide (MTT, Sigma-Aldrich, St. Louis, MO, USA) is reduced in mitochondria by dehydrogenases to purple formazan, which is then dissolved in DMSO. Aliquots of 100 µL of the developed solutions were transferred to corresponding wells of 96-well plates and measured at 560 nm using a plate reader (GloMax TM-MultiDetection system with Instinct Software, Madison, WI, USA). The mean values of 15 measurements were plotted in histograms. The error bars represent the standard deviations.

Similarly, after the treatments (24 h), 10 µL aliquots from the cell culture media were subjected to lactate dehydrogenase assay (LDH, Abcam, Cambridge, UK) according to the supplier protocol. In this assay, LDH as a cytosolic enzyme releases the cells after damage to the plasma membrane. The absorbance of the LDH color assay was measured at 490 nm.

Significant difference values were calculated using the one-way test ANOVA: * *p* < 0.05, ** *p* < 0.01, *** *p* < 0.001.

### 2.5. Flow Cytometric Analysis of Apoptosis and Caspase-3 Level

SKBR3 were collected with trypsin/EDTA (ThermoFisher Scientific) and centrifuged at 600 rpm. The U87MG spheroids were harvested, and the cells were detached with 2.21 mM EDTA. Cell pellets were resuspended in Annexin-V binding buffer (Mitenyi Biotec B.V. & Co. KG, Bergisch Gladbach, Germany) to which AnnexinV/FITC (Mitenyi Bi-otec B.V. & Co. KG, Bergisch Gladbach, Germany) or NucView^®^ 488 caspase-3 substrate was added. Propidium iodide (PI, Mitenyi Biotec B.V. & Co. KG, Bergisch Gladbach, Ger-many) was added to the cell suspension just before detection by flow cytometer (MACSQuant^®^ Analyzer, Miltenyi, Bergisch Gladbach, Germany) in channels B1 and B3. Hypericin fluorescence was detected in the B2 channel.

### 2.6. Singlet Oxygen Phosphorescence and Production Measurements

Label-free technique: The phosphorescence of ^1^O_2_ at 1270 nm was investigated for two different spheroid systems: SKBR3 (~10^6^ cells) and spontaneous U87MG (~10^6^ cells) spheroids, which were added to 2 mL of complete media in a 10 × 10 × 40 mm quartz cuvette equipped with an overhead-type glass stirrer and kept at ~30 °C.

The optical setup was described in our previous work [25]. A laser system consisting of a pulsed optical parametric oscillator (OPO) (GWU basiScan-M, Erfstadt, Germany) pumped with the third harmonic of a Nd:YAG laser (Spectra-Physics, Quanta-Ray, INDI-HG-10S, Milpitas, CA, USA) was used to excite the samples. The laser wavelength was set to 598 nm matching the absorption maximum of hypericin. The repetition rate of the 5–7 ns long laser pulses was 10 Hz. The time course of the singlet oxygen phosphorescence signal was measured in the 1250–1300 nm spectral region using a photomultiplier tube (Hamamatsu H10330A-75, Shizuoka, Japan), operated in photon counting mode. The disturbing background emission originating from the sample fluorescence was suppressed by subtracting the average of two auxiliary measurements (acquired in the adjacent spectral regions) from the measured time course. Special care was taken to avoid detector saturation in all of the measured spectral regions. The average laser power was 60 μW in case of U87 MG and 1mW in case of SKBR3. The signal from 500 consecutive laser pulses was averaged in order to increase signal to noise ratio.

Fluorescent labeling technique: ^1^O_2_ production was imaged (using the microscopy system as described in 2.3) with singlet oxygen sensor green (SOSG, ThermoFisher Scientific) in U87MG cell spheroids before and after irradiation with (i) ^1^O_2_ phosphorescence set-up during ^1^O_2_ measurement at 598 nm (1 h), and (ii) irradiation platform for PDT at 590 nm with 4 and 10 J/cm^2^. Excitation of SOSG was performed with 488 nm laser, and green emission was detected in the spectral region 490–540 nm. The intensity of green fluorescence of SOSG in spheroids was evaluated using ImageJ software based on the images.

Absorption of SKBR3 cells before, immediately after, 1, 2, and 3 h after 5 µM hypericin administration was detected using Specord 600 diode-array spectrometer (Analytik Jena AG, Jena, Germany) at room temperature. UV-Vis absorption spectra were collected every 0.5 nm in the spectral range 400–700 nm.

### 2.7. Ex Ovo CAM Model with Spheroids Preparation

For the preclinical model, a chorioallantoic membrane (CAM) of the embryo of a fertilized Japanese quail (Coturnix japonica) (a breeding colony of IABG SASci) was prepared, as previously reported [6]. On embryonic day (ED) 7 and 9, the U87MG spheroids were transferred to the CAM and incubated at 37 °C and 50–60% relative humidity in the dark until ED11. The silicone ring (⌀ 6 mm) was positioned on the surface of CAM at ED11 to capture the spheroids, and 30 µL of 500 nM and 10 µM hypericin in PBS was administered topically into the ring. Different treatment protocols were applied: Vehicle–DMSO, Vehicle-complete D-MEM, CAM with PBS, CAM with spheroids, CAM with spheroids and hypericin in the dark, CAM with spheroids and irradiation with 405 nm (285 mW/cm^2^) for 4 min, CAM with spheroids and hypericin and irradiation with 405 nm (285 mW/cm^2^) for 4 min. RGB and fluorescence images were acquired with a digital camera (Canon EOS 6D II with Canon MP-E 65mm f/2.8, Tokyo, Japan) before hypericin administration, immediately, 1, 3, 5, and 24 h after administration or PDT. Fluorescence images were taken with the same camera but with violet excitation light (custom-made circular blue LED light with a wavelength of 405 nm). Fluorescence of hypericin was evaluated from the red channel of the RGB images using ImageJ software (National Institutes of Health, USA).

The CAM tissue containing spheroids and hypericin was separated at ED12 and fixed with 4% paraformaldehyde (Sigma-Aldrich, Germany). Then, 30 µm frozen sections were prepared for histopathological analysis to determine the interaction of spheroids with CAM and the photodestruction of the tissue (hematoxylin (BAMED, Malacky, Slovakia) and eosin staining). Sections were examined using a Kapa 2000 light microscope (Kvant, Bratislava, Slovakia) with 10× and 20× objectives and a Nikon E995 digital camera (Nikon, Tokyo, Japan).

### 2.8. PCR Analysis of CAM with Spheroids

CAM tissue with and without spheroids was separated at ED12 and frozen in liquid nitrogen to isolate the total RNA using TRI-Reagent (Molecular Research Center, Cincinnati, OH, USA), according to the manufacturer’s protocol. The RNA was precipitated with isopropanol, washed with 75% ethanol, and dissolved in 50 µL of pure, RNAse-free water. The concentration and purity of the RNA was measured spectrophotometrically using a Multiscan Go (Thermo Fisher Scientific). To exclude contamination with genomic DNA, the RNA samples were treated with DNAse (Thermo Fisher Scientific).

From 2 µg of total RNA, cDNA was synthesized. The reaction mixture (40 µL) contained 2 µg RNA, oligo (dT)18 primer (100µM, Thermo Fisher Scientific), random hexamer primer (100µM, Thermo Fisher Scientific), RT buffer 5× (250 mM Tris-HCl pH 8.3, 375 mM KCl, 15 mM MgCl_2_ containing 0.1 M DTT, Thermo Fisher Scientific), RiboLockRNase Inhibitor (40U/µL, Thermo Fisher Scientific), DNTP Mix (10mM, Thermo Fisher Scientific), and RevertAid Reverse Transcriptase (200U/µL, Thermo Fisher Scientific). The final cDNA was diluted 10 times, and 5 μL aliquot was used for qPCR analysis.

The qPCR primers are listed in Table 1. The final concentration was 0.5 μM for each primer. The qPCR was performed in 20 µL using FastStart DNA Master SYBR Green I (Roche, Basel, Switzerland). Reaction conditions followed the manufacturer’s instructions with annealing at 55 °C.

Transcripts of VEGF-A, Quek1 (quail receptor for VEGF-A), interleukin 8, TLR-7, interferon α, and GAPDH (glyceraldehyde-3-phosphate dehydrogenase) were analyzed using LightCycler^®^ Nano (Roche). Final data were normalized to GAPDH mRNA levels. Control group mRNA levels were set to 1. Alternatively, hypericin treated samples were set at 1 to compare the effect of PDT. One-way rank comparison ANOVA, followed by Dunn’s multiple comparison test was used for statistical analysis. We reported the results as mean ± SD (standard deviation). Analysis was performed using SigmaPlot (Systat Software, San Jose, CA, USA).

## 3. Results

### 3.1. Assesment of ^1^O_2_, Caspase-3, and Apoptosis in 3D Model of SKBR3 Cells as Compact Aggregates

**Figure 1:**
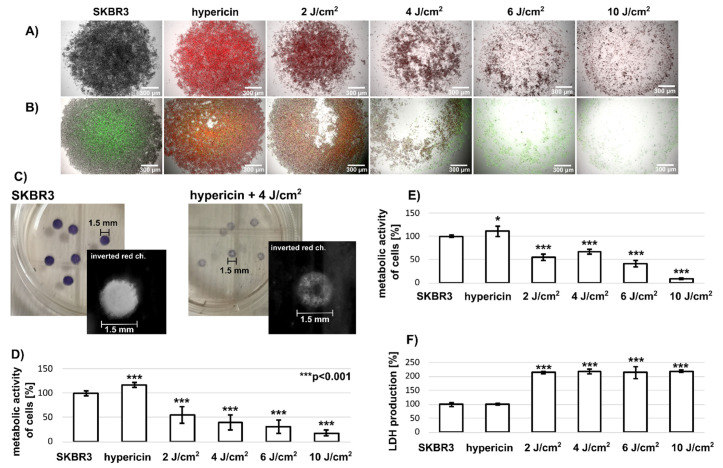
(**A**) Microscopical images of SKBR3 spheroids in the presence and absence of 500 nM hypericin (red fluorescence) and 24 h after application of different light doses 2–10 J/cm^2^. Scale bar represents 300 µm. (**B**) Caspase-3 levels in these cells detected with NucView^®^488 Caspase-3 substrate. Scale bar represents 300 µm. (**C**) Representative images of SKBR3 spheroids after MTT application (case without treatment and after 4 J/cm^2^). The black and white images represent inverted red channels of RGB images. Scale bar represents 1.5 mm. (**D**) Analysis of the intensities of white circles (related to formazan production in the cells) shows the metabolic activity of cells in spheroid. (**E**) Metabolic activity of cells in spheroids measured by dissolving formazan in DMSO. (**F**) LDH production of SKBR3 cells in spheroids. The degree of significant difference was analysed by one-way method ANOVA, * *p* < 0.05 and *** *p* < 0.001. All experiments were performed in triplicates.

SKBR3 spheroids were grown in hanging drops. However, after transfer to a Petri dish, the spheroids adhered to the surface and formed compact aggregates. We refer to this model as superficial tumors. Fluorescence of hypericin in SKBR3 cells in the form of compact aggregates incubated with 500 nM hypericin for 3 h and detected 24 h after removal of hypericin from the medium showed a bright red color (Figure 1A). Irradiation of these cells with light (2–10 J/cm^2^) resulted in a decrease in fluorescence and fragmentation of the compact aggregates. A small aggregate fraction with a visible corona can still be seen after application of a light dose of 10 J/cm^2^. Caspase-3 levels in these cells were detected using the NucView^®^488 Caspase-3 substrate, which is shown with green fluorescence in Figure 1B. It can be seen that the central part of the control spheroid is already rich in caspase-3 and can respond positively to apoptosis in these control cells.

Phototoxicity of PDT to cells in this formation was determined by adding MTT to the cell culture medium. Compact aggregates of SKBR3 cells were highlighted by a purple color in Figure 1C. Donut-shaped aggregates were identified after a light dose of 4 J/cm^2^. Phototoxicity associated with metabolic activity of these cells was assessed using inverted 8-bit grayscale images (Figure 1C: black and white insets from each aggregate). This method revealed a decreasing metabolic activity of cells after PDT (2–10 J/cm^2^) (Figure 1D). The dissolution of formazan crystals in SKBR3 aggregates (Figure 1E) resulted in a similar effect as image analysis. In addition, LDH assay was performed to determine the damage to the cell plasma membrane caused by PDT. As expected, LDH production by SKBR3 cells in aggregates increased significantly after PDT (Figure 1F). Similar LDH levels were observed in all PDT groups regardless of the light doses studied.

**Figure 2:**
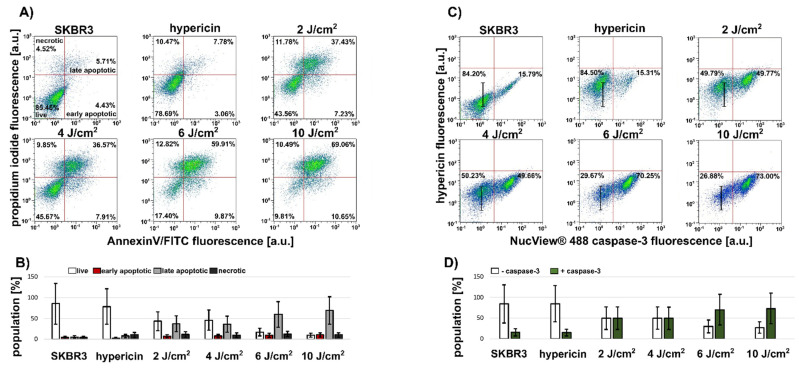
(**A**,**B**) Flow cytometric analysis of apoptotic (AnnexinV/FITC positive) and necrotic (propidium iodide positive) cell populations triggered in SKBR3 spheroids in the presence and absence of 500 nM hypericin and 24 h after application of different light doses 2–10 J/cm^2^. (**C**,**D**) Flow cytometric analysis of caspase-3 by NucView^®^488 caspase-3 substrate and hypericin (intensity shift is denoted with black marker) fluorescence detection in cells of SKBR3 spheroids. Cell number is color coded from blue (minimum) to red (maximum). Error bars are standard deviations of measurements. Experiments were performed in duplicate. Quadrants in A were selected to distinguish live (white columns), early apoptotic (red columns), late apoptotic (grey columns), and necrotic (dark grey columns) cells. Quadrants in (**C**) were selected to distinguish caspase-negative (left quadrants and white columns) and caspase-positive (right quadrants and green columns) cells.

The heterogeneous distribution of caspase-3 in SKBR3 spheroids was clearly evident from the images. In addition, flow cytometry was used to determine different cell populations with increased caspase-3 content and apoptosis positivity. The use of trypsin/EDTA prior to flow cytometric analysis makes it very easy to separate the compact SKBR3 aggregates into single cells available for detection. For this reason, caspase-3 levels and apoptosis were examined in the separated cells. Figure 2A shows the heterogeneity and increase in apoptotic and necrotic cell populations. The application of 10 J/cm^2^ resulted in 10% early apoptotic, 69% late apoptotic, and 10% necrotic cells (Figure 2B). This treatment resulted in the identification of 73% caspase-3-positive cells (Figure 2C,D). A visible reduction in hypericin fluorescence (black marker in Figure 2C) was observed in caspase-3-negative cells (Figure 2D). The population of caspase-3-positive cells increases with the increasing numbers of apoptotic cells and an applied light dose. Consistent with caspase-3 imaging, 16% of the caspase-3-positive cells were identified in SKBR3 control spheroids (Figure 2C).

**Figure 3:**
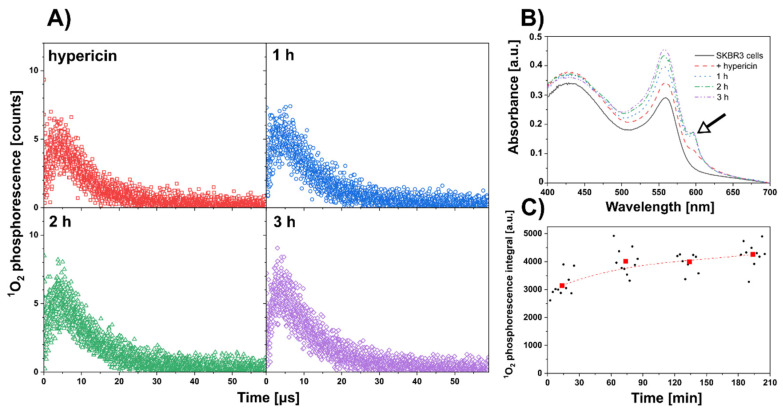
(**A**) Singlet oxygen (^1^O_2_) phosphorescence produced in the solution of 5 µM hypericin and SKBR3 cells measured immediately (red), 1 h (blue), 2 h (green), and 3 h (violet) after hypericin administration. (**B**) Absorbance spectra of SKBR3 cells detected before (black), immediately (red), 1 h (blue), 2 h (green), and 3 h (violet) after hypericin administration. Black arrow indicates hypericin absorption. (**C**) Integral of ^1^O_2_ phosphorescence determined at each acquisition point (black circles–particular values, red square–average of 10 particular values). The experiments were repeated at least twice.

Flow cytometry showed that the cells in the central part of the SKBR3 spheroid were already distinct from the cells in the periphery. For this reason, it was of great interest to see if we could generate ^1^O_2_ from hypericin absorbed in these cells. In the next experiment, we demonstrated the formation of ^1^O_2_ in cells from hypericin using our label-free approach. The time-course of ^1^O_2_ phosphorescence (following pulsed excitation) was detected in cells of the SKBR3 aggregate incubated with 5 µM hypericin for 0–3 h. Note that these cells were disaggregated during the measurements. The kinetics of ^1^O_2_ phosphorescence for each time point after hypericin administration is shown in Figure 3A. The corresponding absorption spectra of hypericin in solution with SKBR3 cells show an increase in the range of 560–600 nm (absorption bands of hypericin) due to hypericin internalization in the cells as shown in Figure 3B. The fluorescence image of hypericin in these cells taken after ^1^O_2_ measurement shows a homogeneous distribution of hypericin in the cells except for the nuclei (data not shown). No hypericin fluorescence was detected in the extracellular medium. The total (integrated) ^1^O_2_ phosphorescence signal gradually increases over the 3 h (Figure 3C).

### 3.2. Assesment of ^1^O_2_ Production, Elevation of Caspase-3 and Apoptosis in Spontaneously Formed 3D Model of U87MG Cells

**Figure 4:**
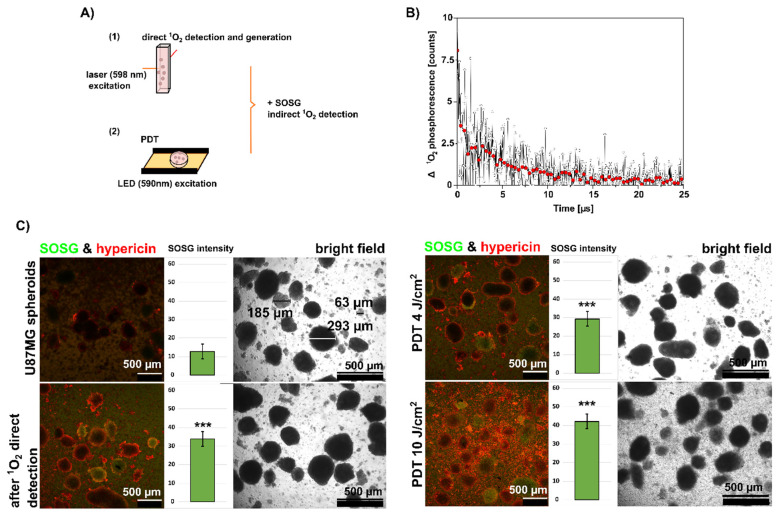
(**A**) Schematic illustration of the approaches used for the generation of ^1^O_2_ (1 and 2) and the detection of ^1^O_2_ by direct and indirect methods. (**B**) Difference in ^1^O_2_ phosphorescence in cells with and without 5 µM hypericin detected by the direct approach (1st in **A**). Raw data are presented in black, and mean values in red. (**C**) Bright field and fluorescence microscopical images of U87MG cells that spontaneously formed spheroids with 500 nM hypericin (red fluorescence), before and after direct ^1^O_2_ detection and after irradiation with 590 nm at 4 and 10 J/cm^2^ (generation of ^1^O_2_ with the second approach shown in (**A**). Scale bar represents 500 µm. Singlet Oxygen Sensor Green (SOSG) was used to detect ^1^O_2_ production in the cells (green fluorescence). The green histograms in the insets show the mean SOSG intensity determined from the fluorescence images. Experiments were performed in duplicate. The degree of significant difference was analysed using the one-way method ANOVA, *** *p* < 0.001.

In contrast to SKBR3, U87MG cells can spontaneously form spheroids of uneven size when cultured for an extended period (2 weeks in the same flask). A representative image of these spheroids (63–293 µm in diameter) is shown in Figure 4. ^1^O_2_ production by hypericin was detected in these spheroids by two methods: direct time-resolved measurements of ^1^O_2_ phosphorescence (Figure 4A,B) and indirectly by administration of a SOSG fluorescent probe (Figure 4A,C). In addition, two approaches to ^1^O_2_ generation were used in these measurements: laser excitation at 598 nm in the ^1^O_2_ phosphorescence detection setup, and LED excitation at 590 nm, which was used in PDT.

For ^1^O_2_ detection in spheroids using the time-resolved method, the hypericin concentration was increased to 5 µM. In general, the ^1^O_2_ phosphorescence signal is extremely weak. Moreover, the ^1^O_2_ emission at 1270 nm is overlapped with a fluorescence and phosphorescence signals originating from the cells and possibly also from the photosensitizer. This background was especially strong in the U87MG cells. In this case, to prove the presence of singlet oxygen originating from hypericin, it was necessary to subtract the background signal (cells without hypericin) from the data acquired with cells containing hypericin. The result is shown in Figure 4B.

Compared with SKBR3 in the spheroids of U87 MG, the ^1^O_2_ phosphorescence signal was low. The fluorescent background saturating the detector did not allow us to increase the laser power on the sample. As mentioned earlier, two control measurements were performed to prove that the late phosphorescence signal was from ^1^O_2_ generated by hypericin in the cells. First, the spheroids without hypericin were irradiated with the same experimental setup, and the measured signal was subtracted from the phosphorescence of the cells loaded with hypericin. These data are shown in Figure 4B. Second, after irradiation of the hypericin-containing spheroids, the sample was centrifuged, and the supernatant was analyzed for residual singlet oxygen phosphorescence originating from hypericin in the medium. No late phosphorescence was observed in the supernatant.

Since we used 500 nM hypericin in the further experiments, the SOSG signal was measured in spheroids containing 500 nM hypericin (red color in Figure 4C). The increase in green fluorescence of SOSG after PDT (4 and 10 J/cm^2^) is shown in Figure 4C. However, a significant increase in SOSG was also observed in spheroids after time-resolved measurements (with pulsed irradiation) performed in spheroids containing 500 nM hypericin. This change can be observed as an increase in green fluorescence inside the spheroids (green histograms).

**Figure 5:**
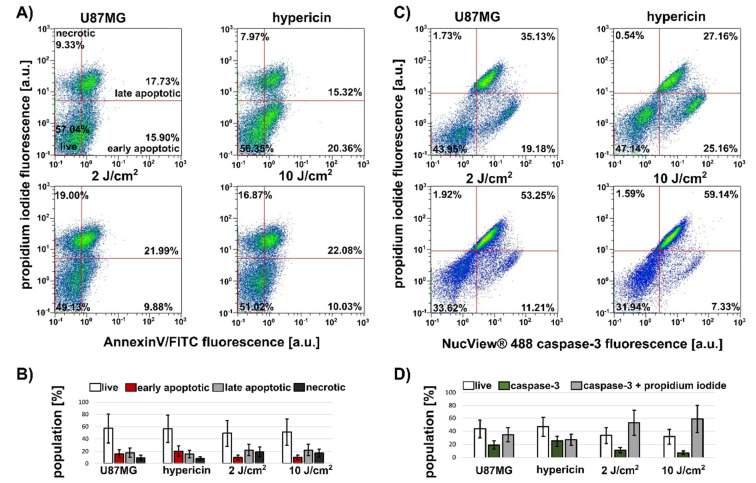
Flow cytometric analysis of propidium iodide and (**A**) AnnexinV/FITC and (**C**) NucView^®^488 caspase-3 fluorescence in cells derived from spheroids of U87MG cells detected under different treatment conditions: 500 nM hypericin in the dark and PDT with light doses of 2 and 10 J/cm^2^. Cell numbers in the graphs are color-coded from blue (minimum) to red (maximum). (**B**,**D**) Population in % corresponding to live cells (white columns), early apoptosis (red columns), late apoptosis (grey columns), necrosis (dark grey columns), caspase-3-positive (green columns), and caspase-3 + propidium iodide-positive (grey columns) cells. Experiments were performed in duplicate. The error bars represent the standard deviation of the measurements.

It would be interesting to see whether the ^1^O_2_ produced in the U87MG spheroids, which is much weaker than in the SKBR3 spheroids, can produce a significant PDT effect. Greater heterogeneity of cell populations was observed in the control spheroids (Figure 5A,C). We adjusted the four quadrants to separate propidium iodide-positive and -negative cells. In addition, early and late apoptotic cells were labeled with AnnexinV/FITC, as shown in Figure 5A.

Apoptotic and necrotic cell populations can already be detected in the control spheroids. With the increasing PDT light dose, the population of “live” cells (lower left quadrant) decreases, and the population of apoptotic (lower and upper right quadrant) and necrotic cells (upper left quadrant) increases (Figure 5A,B). In fact, we still detected 50% “live” populations. This may be due to less light damage in U87MG spheroids than in SKBR3 spheroids.

Similarly, U87MG spheroids labeled with propidium iodide and NucView^®^488 caspase-3 showed higher “live” populations in control spheroids, and more caspase-3-positive and propidium iodide-positive cells were detected after PDT (Figure 5C,D). More than 50% of cells were positive for caspase-3 and propidium iodide in samples at 2 and 10 J/cm^2^.

**Figure 6:**
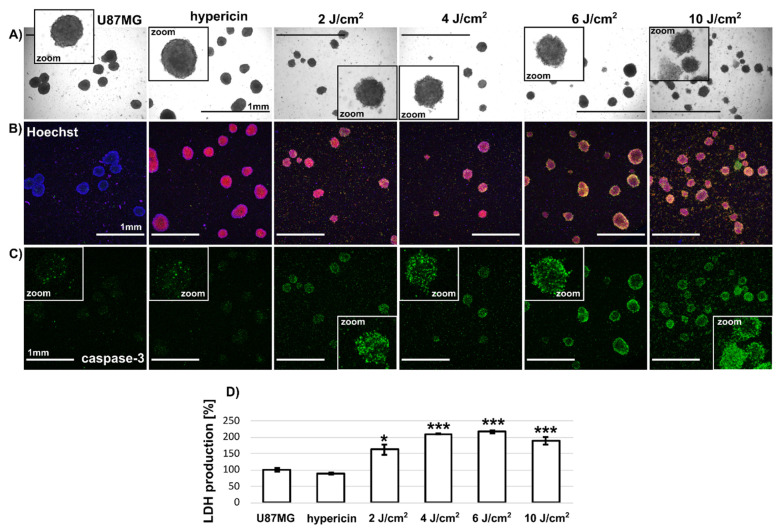
(**A**) Brightfield and fluorescence (**B**) overlap images of Hoechst (blue), hypericin (red) and (**C**) NucView^®^488 caspase-3 (green). The insets show magnifications to better identify the morphology of the spheroids. U87MG cells spontaneously formed in spheroids were detected in the absence and presence of 500 nM hypericin and 24 h after irradiation with different light doses (2–10 J/cm^2^). Scale bar represents 1 mm. (**D**) LDH production in U87MG cells without and with 500 nM hypericin and irradiation (2–10 J/cm^2^). The level of significant differences was analysed by one-way method ANOVA, * *p* < 0.05 and *** *p* < 0.001. The experiments were performed in triplicate.

We have demonstrated heterogeneity in the cell populations of U87MG spheroids using flow cytometry. For this reason, we focused our attention on imaging this heterogeneity. The morphology of U87MG spheroids spontaneously formed and treated with 500 nM hypericin and PDT (2–10 J/cm^2^) is shown in Figure 6A. The insets of the brightfield images show changes in size, decreasing on average from 150 to 100 µm (see also Table 2), and a change in the shape of the spheroids (at higher light doses, single cells can be seen detaching). In the corresponding fluorescence images, the nuclei (Hoechst-blue), hypericin distribution (red) and caspase-3 positive cells (NucView488 caspase-3-green) were observed in the spheroids. An overlap of this distribution can be seen in Figure 6B. After PDT, an increase in caspase-3-positive cells was observed in the cells, most markedly after the application of 10 J/cm^2^ (Figure 6C). Many single cells released from the spheroids were also caspase-3 positive. As expected, few caspase-3-positive cells (green color) were observed in the control spheroids (Figure 6C). In contrast, a homogeneous distribution of hypericin (red color) and Hoechst, which was localized in the cell nuclei (blue color), was observed (Figure 6B). However, this is consistent with the flow cytometry results in Figure 5C.

LDH production was detected in the culture media of the spheroids. Because it is related to plasma membrane damage, we wanted to observe the LDH production rate as a function of PDT of U87MG spheroids. It can be seen that LDH production in the spheroids treated with PDT increased with the applied light dose (Figure 6D). However, the damage (LDH level) appears to be similar at higher light doses (>4 J/cm^2^).

### 3.3. Assesment of Caspase-3 and LDH Level in U87MG Spheroids-Grown by Hanging Drop Method

**Figure 7:**
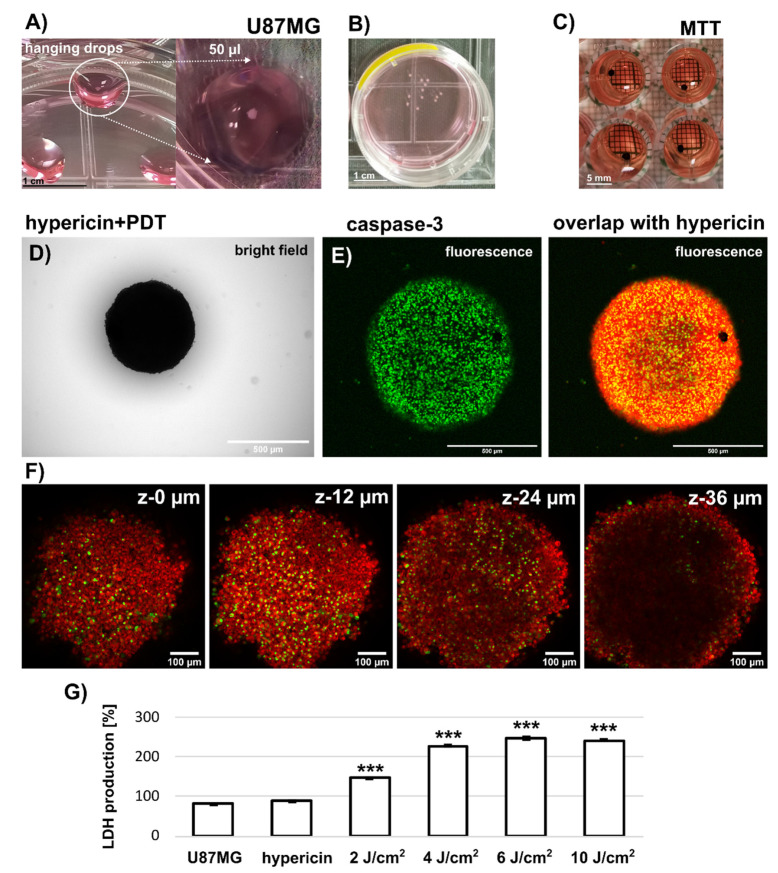
(**A**,**B**) Representative images of U87MG spheroids grown by the hanging drop method. Scale bar represents 1 cm. (**C**) Representative image of purple formazan formed in the spheroids. Scale bar represents 5 mm. (**D**) Bright field (Scale bar represents 500 µm). and (**E**) fluorescence images of U87MG spheroids incubated for 3 h with 500 nM hypericin (red fluorescence) and irradiated at 4 J/cm^2^. Caspase-3 (green) was detected 24 h after irradiation. Scale bar represents 500 µm. (**F**) Z-stack detection of fluorescence in the spheroid. Scale bar represents 100 µm. (**G**) LDH production in U87MG spheroids without and with 500 nM hypericin and irradiation (2–10 J/cm^2^). The degree of significant difference was analysed by one-way method ANOVA, *** *p* < 0.001. The experiments were performed in triplicate.

The third type of spheroids were large (>500 µm) U87MG spheroids grown by the hanging drop method (see Figure 7A). These spheroids were placed in Petri dishes to reach a size of 1 mm in diameter (Figure 7B). An assessment of phototoxicity and metabolic activity in these spheroids by MTT could not be performed, because the formazan produced by the spheroids was firmly entrapped (see Figure 7C). It could not be readily dissolved in DMSO to produce a confluent signal for absorption detection by the plate reader. In addition, the image analysis (similar to Figure 1) failed to detect differences after PDT (2–10 J/cm^2^) compared with the control (data not shown). We next focused on fluorescence imaging of these large spheroids.

Figure 7D shows a representative bright field image of the U87MG spheroid. The fluorescence of hypericin (red) and NucView488 caspase-3 (green) detected in this spheroid is shown in Figure 7E. However, detection by confocal fluorescence microscopy reaches the optical limits due to the size of the spheroids. As shown in Figure 7F, we can detect fluorescence signals from the peripheral cell layers, whereas the core of the spheroid is “blind” for our technique. Similar to spontaneously formed U87MG spheroids, the LDH production increased significantly in large U87MG spheroids treated with PDT (2–10 J/cm^2^). Differences were also observed in morphology, and the size of spheroids decreased with applied light doses from 860 to 540 µm in diameter (Table 2).

### 3.4. Applicability of Large U87MG Spheroids for In Vivo Photodiagnostic Applications on Quail CAM

**Figure 8:**
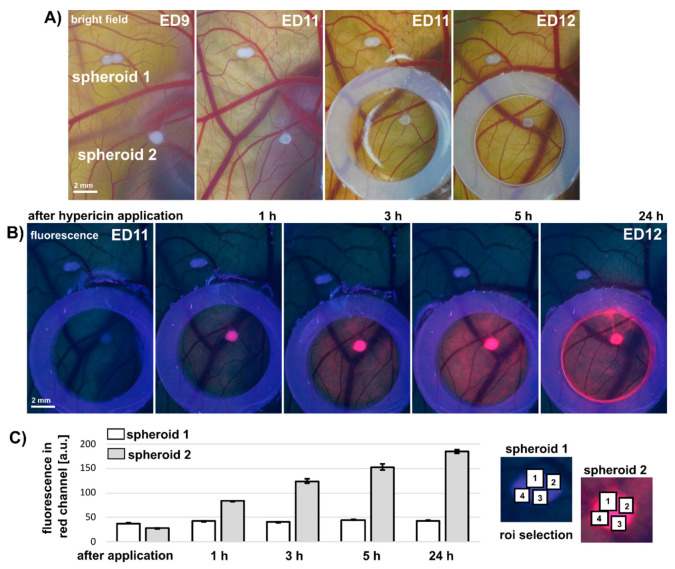
(**A**) Representative color images of quail CAM with U87MG spheroids detection right after and 1–3 days after the implantation. Silicon ring and hypericin was applied at 2nd day after spheroids implantation. Scale bar represents 2 mm. (**B**) Fluorescence of 10 µM hypericin (pink) detected in spheroid 2 right after and 1, 3, 5 and 24 h after hypericin administration. Scale bar represents 2 mm. (**C**) Fluorescence intensity detected in four spots (as illustrated in right) of spheroid 1 and 2 from red channel of RGB images. The error bars represent the standard deviation of the measurements. Experiment was repeated at least four times.

The size of the U87MG spheroids allows them to be transferred to the preclinical model of the quail chorioallantoic membrane (CAM) to create in vivo conditions. Thanks to the large volume, it is possible to see the spheroids even with the naked eye. An example of this model and the spheroids applied to it is shown in Figure 8. Bright field RGB color images of CAM were acquired in white light on ED9-ED12 (Figure 8A). Two areas were selected: spheroid 1 and spheroid 2. A silicone ring was placed around spheroid 2, and hypericin solution was administered only into this area. Fluorescence pharmacokinetics (Figure 8B) was detected in ultraviolet light (420 nm) for 0, 1, 3, 5, and 24 h. The development of pink hypericin fluorescence can be observed only within the silicon ring and mainly in spheroid 2 (Figure 8B,C). Note that the hypericin concentration in this experiment was 10 µM. It can be seen that photodiagnosis of cancer cells can be performed within 1 h after topical administration of hypericin.

**Figure 9:**
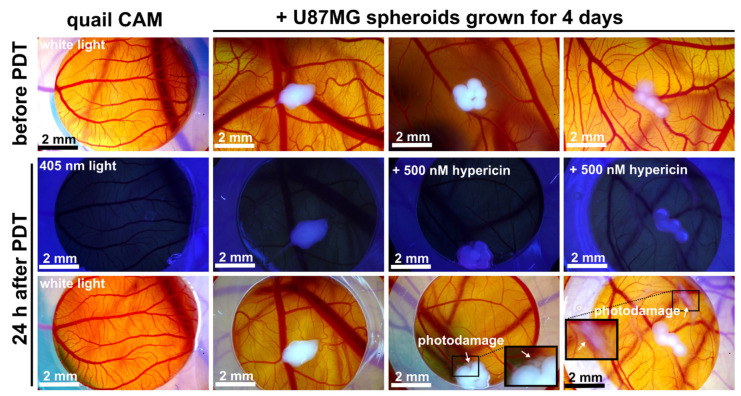
Representative color images of quail CAM without and with U87MG spheroids grown for 4 days and detected before PDT and hypericin administration (top panel). Fluorescence of CAM without and with spheroids, and without and with 500 nM hypericin (administered 3 h before PDT) was detected 24 h after PDT application (middle raw image). The last raw image corresponds to the color images of the same CAMs in white light. Insets are zoomed areas of photodamage caused by PDT. Experiment was repeated at least four times. Scale bar represents 2 mm.

Further, for PDT, the hypericin concentration was decreased to 500 nM as previously used in vitro. Figure 9 shows illustrative images before application of the spheroids, 4 days after their application at CAM, and after PDT with a 405 nm laser light (4 min, 285 mW/cm^2^). The fluorescence images do not show pink fluorescence due to the low hypericin concentration (Figure 9). However, two effects can be observed after PDT: (1) peripheral cells of the spheroids were detached and released from the spheroid (see inset), (2) photodamage of the cells in the area of hypericin application was observed (see inset in lower right image). No significant difference in spheroid size was observed at this dose compared to the untreated control (Figure 9).

**Figure 10:**
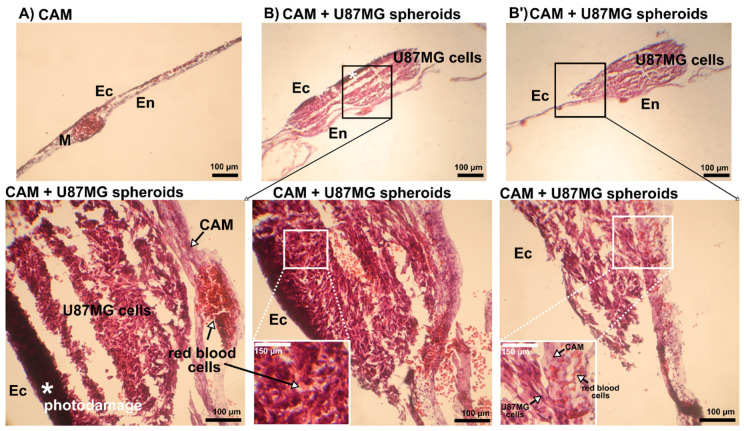
Representative H&E histology section of CAM with U87MG spheroids treated with 500 nM hypericin. (**A**) Region of CAM without spheroids (Ec-ectoderm, En-endoderm, M-mesoderm), and (**B**,**B’**) two different views of CAM with U87MG cell spheroids. The second row of the panel shows zoomed regions of interest indicated by black rectangles in (**B**,**B’**) rotated in position from En in left. Attachment of U87MG cells to CAM is visible. White asterisks * indicate photodamage to U87MG cells. The position of CAM, red blood cells, and U87MG cells in each section is indicated with a black arrow. The photodamage was caused by the light coming from the detection system during imaging. Scale bar represents 100 µm.

Histology of CAM with spheroids, hypericin administration, and irradiated only with photographic acquirement is shown in Figure 10. CAM without spheroids is shown in Figure 10A and U87MG cell spheroids are shown in Figure 10B. The cavities in the spheroids are due to the preparation technique. The invagination of the spheroidal cells into the ectoderm of CAM can be seen in the zoom images of Figure 10B,B’. Light damage caused by irradiation with the detection system was also observed, but only in the first cell layers of the spheroid (dark areas with asterisks).

**Figure 11:**
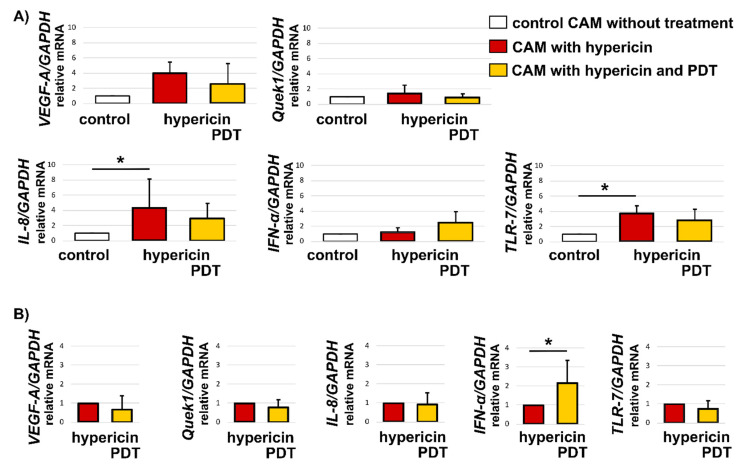
Relative expression of *VEGF-A*, *Quek1*, *IL-8*, *IFN-α*, *TLR-7* normalized to the *GAPDH* gene from CAM without spheroids and hypericin application (control, white columns), with 500 nM hypericin in the dark (red columns), and with hypericin and PDT application (yellow columns). Reference in (**A**) was an untreated control and (**B**) hypericin in the dark. Error bars represent standard deviations from the mean (n = 4–5). The degree of significant difference was determined using the one-way test ANOVA: * *p* < 0.05.

Although macroscopic changes were not observed after PDT, PCR revealed some significant differences in the expression of genes related to the body’s immune response. In the case of *IL-8* and *TLR-7*, higher expression of the mentioned genes was observed after the application of hypericin, especially in the case of hypericin in the dark. After PDT the changes were not significant compared with the control group (Figure 11A).

There were no significant changes in the expression of *VEGF-A* and *Quek1* 24 h after treatment (Figure 11A), but after the application of spheroids, we saw an increase compared with the tissue without spheroids (data not shown).

When hypericin in the dark was considered as a reference (relative mRNA = 1), we also observed the effect of PDT on *IFN-α* gene expression compared with the application of hypericin in the dark (Figure 11B).

## 4. Discussion

In recent decades, many efforts have been made to develop 3D models to replicate the microenvironment of a tumor [18,26,27,28,29]. In the present work, three morphologically distinct types of cancer cell spheroids were produced. The method we used here was a simple hanging drop method in which the cancer cells grew without the addition of growth factors. We showed here that SKBR3 cells were unable to form a spheroid shape, while U87MG cells formed spontaneous and forced spheroids very easily. This provides us with several options for modeling.

SKBR3 cells formed a spatial and superficial model in which cells are tightly connected in the center of the circle and the density of cells decreases toward the periphery. This formation may lead to a decrease in nutrient distribution in the center, which in turn may lead to pathological changes. In the present work, it was shown that the cells in the center of SKBR3 spheroids/aggregates have higher caspase-3 levels (Figure 1B). To our knowledge, this is the first time that the NucView^®^ 488 caspase-3 substrate has been used to visualize caspase-3 in a living 3D system after PDT. Since this model has large dimensions but not a large volume, it is well suited for microscopic observation. We found homogeneous localization of hypericin in the cells of SKBR3 spheroids. However, heterogeneity of cell populations in response to PDT was detected (Figure 2). We hypothesize that this heterogeneity in spheroid response could be caused by a heterogeneous distribution of caspase-3 in untreated spheroids. This basal level could increase PDT efficacy in affected cells.

Confocal fluorescence imaging revealed the homogeneous distribution of hypericin in two of the three 3D systems (Figure 1, Figure 6 and Figure 7). Indeed, hypericin-induced PDT has been studied in several spheroidal 3D models of cancer cells. Bassler et al. recently reported a gradient of hypericin fluorescence lifetime distribution in U87MG spheroids, possibly related to pH and metabolic changes [30]. However, several groups have observed heterogeneous hypericin localization in 3D spheroids. Hempfling et al. demonstrated high peripheral fluorescence of hypericin in rhabdomyosarcoma spheroids [17]. Application of PDT to these cells resulted in a strong decrease in Ki-67 (related to proliferation) and an increase in cleaved caspase-3 in rhabdomyosarcoma spheroids detected by immunostaining. In our study, morphologic changes were observed in the spheroids after PDT (Figure 6 and Table 2). Very often, the damaged cells detached and floated in the medium. We also observed detachment of cells after PDT (Figure 1 and Figure 6). However, the center of the aggregate was initially destabilized only in SKBR3 spheroids. Cells that survived PDT and still adhered to the surface had high levels of caspase-3 (Figure 1B). In all spheroid models, cells exposed to PDT produced high levels of lactate dehydrogenase (Figure 1, Figure 6 and Figure 7), and some of them were positive for apoptosis. It should be noted that the heterogeneity induced in the spheroids could be caused, in particular, by the distribution and penetration of hypericin into the spheroids. Foglietta et al. demonstrated morphological changes in HT-29 spheroids after hypericin, PDT and sonodynamic treatment [31]. Peripheral localization of hypericin was also demonstrated in the work of Vandepitte et al. in which polyvinylpyrrolidone-hypericin was applied to human urothelial and T24 transitional spheroids [32]. In addition to this localization, significant PDT efficacy was also observed. Similar results on the biodistribution of hypericin in spheroids were previously observed in the work of Huygens et al. [19].

It is well known that the induction of apoptosis in PDT is mainly caused by the generation of reactive oxygen species [33]. Hadjur et al. observed the oxidation of lipids in melanoma cells after hypericin PDT [34]. Xu et al. reported the production of nitric oxide and hydrogen peroxide by hypericin in plant cells [35]. Diwu and Lown observed the formation of semiquinone radicals, ^1^O_2_ and superoxide radicals in hypericin solutions [36]. Thomas et al. observed the production of ^1^O_2_ by tetramethylethylene in isolated mitochondria [37]. Our group had previously observed that hypericin produced ^1^O_2_ in solutions of acetone and dimethyl sulfoxide [9,38]. Therefore, we felt compelled to investigate ^1^O_2_ production in aggregates of SKBR3 cells exposed to hypericin using the label-free technique (Figure 3). Phosphorescence of ^1^O_2_ was detected in SKBR3 cells immediately after hypericin administration, and was still detectable 3 h after hypericin incubation with the cells.

Similar to SKBR3 cells, phosphorescence of ^1^O_2_ generated by hypericin was also observed in U87MG spheroids (Figure 4B). Fluorescence imaging of SOSG showed that the most affected cells were located in the periphery of the spheroids (Figure 4C). This is the area where higher fluorescence of hypericin was detected. Such peripheral localization of hypericin was also observed in larger spheroids formed by the hanging-drop method in this study (Figure 7). SOSG was used in our study only to detect areas of ^1^O_2_ generation in spheroids. These results are only complementary to the label-free approach of ^1^O_2_ detection. One should be aware that SOSG is not an ideal probe due to limits in its specificity and penetration into cells [39,40].

We observed an increase in caspase-3 in live U87MG spheroids that depended on the light dose used during PDT (Figure 5). Three cell populations were detected in these spheroids: live cells that reacted negatively to the NucView^®^ 488 caspase-3 substrate and propidium iodide, and two that reacted positively to the NucView^®^ 488 caspase-3 substrate, one of which also reacted to propidium iodide. The third population was preferentially detected at 10 J/cm^2^ PDT (Figure 5C), which was also apoptosis positive. These cells produced significantly higher levels of lactate dehydrogenase (Figure 6). Detachment of the cells was observed at high light doses. Sirenko et al. detected different phenotypes in HCT116, DU145, and HepG2 cancer spheroids, which were recognized by the detection of caspase-3 and the induction of apoptosis [27]. These observations suggest a different behavior of the cells in the spheroids, which may respond differently to PDT. We hypothesize that photodamage induced by PDT could trigger additional cell signaling in the periphery of the spheroid. The second messengers could then transmit further signals to cells in the core of the spheroid, which then trigger the death stimulus.

All models presented have been shown to respond dynamically to PDT. However, we produced the spheroids from a monoculture of cancer cells. Our goal was to create a simple, valuable model of cancerous and non-cancerous tissue suitable for photodiagnistics (especially glioblastoma). We created a CAM model with spheroids grafted onto the ectoderm to compare a noncancerous and a cancerous environment. The specificity of hypericin against cancer cells after its topical administration was observed shortly (1 h) after application (Figure 8). This specificity can persist over a longer period of up to several hours (24 h). Therefore, this model is suitable for the specific administration of hypericin, as has been shown previously with non-spheroidal cells grafted to CAM [5,6]. The application of cancer cell spheroids to quail CAM may affect their vasculature and especially angiogenesis after PDT, as reported by Majernik et al. [41]. Our model could be improved in the future, and may be suitable for angiogenesis studies in healthy and cancerous environments.

The growth of U87MG spheroids on CAM forced infiltration of cancer cells into the ectoderm of CAM. Our preliminary results show that *VEGF-A* and *Quek1* genes were not significantly affected by spheroids and 500 nM hypericin treatment, we only saw a trend caused by spheroid application. The inflammatory genes *IL-8* and *TLR-7* increased significantly after spheroid and hypericin administration. PDT of CAM reduced these genes to baseline levels. Interestingly, the gene expression of *IFN-α* increased after PDT compared with hypericin treatment in the dark. We expected an increase in the expression of *VEGF-A* and *Quek1* after PDT [41,42,43]. However, we used a rather low hypericin concentration for in vivo in our protocols. For this reason, we assume that a higher hypericin concentration should be used to observe a significant effect after PDT. Indeed, hypericin increased the expression of inflammation-responsive genes in the dark. In addition to hypericin, human glioblastoma spheroids were also used, which could induce the observed effect by foreign cells [44,45]. These preliminary results demonstrate the applicability of the glioblastoma spheroid model to quail CAM, and the effect of hypericin and PDT treatment on gene expression in CAM due to their presence.

## 5. Conclusions

We have presented three models of spheroids, in which caspase-3 can be monitored by NucView^®^ 488 caspase-3 substrate detection with confocal fluorescence microscopy and flow cytometry. In addition, the large U87MG spheroids grown in hanging drops can be effectively transplanted at CAM to monitor cancer cell targeting by photosensitizers and photodiagnostics. The main objective of the present study was to highlight the experimental limitations arising from the 3D structure of the spheroid models. This affected the imaging and visualisation of the spheroids in photodiagnostics. In live 3D spheroids, changes in caspase-3 distribution were detected during PDT with hypericin, which correlated with the heterogeneity of cell populations observed by flow cytometry. However, this evidence is limited by the size of the spheroid. Furthermore, we have shown that a significant level of caspase-3 is maintained in untreated spheroids. However, this level can be increased during PDT by the formation of reactive oxygen species and, in particular, ^1^O_2_, which has been shown to be formed in living cells of hypericin-loaded spheroids. The efficacy of PDT in spheroids also depends on the depth of hypericin accumulation and the applied light dose. Importantly, we have established a model of glioblastoma spheroids on the quail model CAM that can be used in future PDT studies. In this model, the induction of *IL-8*, *TLR-7*, and *IFN-α* by hypericin administration was demonstrated. The mechanism of PDT in each layer of spheroids should be better studied. However, we have shown that the preclinical model of glioblastoma spheroids at CAM is a suitable model for screening drugs, delivery systems, and therapies.

## Figures and Tables

**Table 1:** The primer sequences and amplified characteristics.

Gene	Primers (5′-3′)	T_m_ (°C)
*VEGF-A*–for1	CGG AAG CCC AAT GAA GTT ATC	59.4
*VEGF-A*–rev1	GCA CAT CTC ATC AGA GGC ACA	64.0
*Quek1*-for1	CAT CAA TGC GAA TCA TAC AGT TAA G	60.9
*Quek1*–rev1	CAT TCA CAA GCA GGG TGA ATG	59.4
*IL-8–*for1	CTG AGG TGC CAG TGC ATT AG	63.5
*IL-8–*rev1	AGC ACA CCT CTC TTC CAT CC	63.3
*TLR-7*–for1	AGA TGT TTT CTG GGC AGA CG	60.0
*TLR-7*–rev1	AAT GAC TTC AAC CGG TTA CTG G	60.0
*IFN*-*α–for1*	CCT TGC TCC TTC AAC GAC A	64.1
*IFN*-*α–rev1*	CGC TGA GGA TAC TGA AGA GGT	62.3
*GAPDH*–for1	GAA CGC CAT CAC TAT CTT CCA G	62.1
*GAPDH*–rev1	GGG CTG AGA TGA TAA CAC GC	60.5

**Table 2:** Reduction in size of U87MG spheroids observed after hypericin mediated PDT. The values represent the average size values of all spheroids detected (n = 15).

Protocol	Hanging Drop Method	Spontaneous Formation
untreated control	860 ± 100 µm	150 ± 30 µm
hypericin	780 ± 50 µm	150 ± 30 µm
+2 J/cm^2^	740 ± 40 µm	120 ± 20 µm
+4 J/cm^2^	670 ± 70 µm	120 ± 30 µm
+6 J/cm^2^	600 ± 80 µm	110 ± 40 µm
+10 J/cm^2^	540 ± 50 µm	100 ± 20 µm

## Data Availability

Data are available in the manuscript.
